# Real-Time Dynamic Adsorption Processes of Cytochrome *c* on an Electrode Observed through Electrochemical High-Speed Atomic Force Microscopy

**DOI:** 10.1371/journal.pone.0116685

**Published:** 2015-02-11

**Authors:** Kouta Takeda, Takayuki Uchihashi, Hiroki Watanabe, Takuya Ishida, Kiyohiko Igarashi, Nobuhumi Nakamura, Hiroyuki Ohno

**Affiliations:** 1 Department of Biotechnology and Life Science, Tokyo University of Agriculture and Technology, Tokyo, Japan; 2 Department of Physics, Kanazawa University, Kakuma-machi, Kanazawa, Japan; 3 Department of Biomaterials Sciences, Graduate School of Agriculture and Life Sciences, The University of Tokyo, Bunkyo-ku, Tokyo, Japan; 4 Advanced Low Carbon Technology Research and Development Program (ALCA), Japan Science and Technology Agency (JST), Tokyo, Japan; US Naval Reseach Laboratory, UNITED STATES

## Abstract

An understanding of dynamic processes of proteins on the electrode surface could enhance the efficiency of bioelectronics development and therefore it is crucial to gain information regarding both physical adsorption of proteins onto the electrode and its electrochemical property in real-time. We combined high-speed atomic force microscopy (HS-AFM) with electrochemical device for simultaneous observation of the surface topography and electron transfer of redox proteins on an electrode. Direct electron transfer of cytochrome *c* (cyt *c*) adsorbed on a self-assembled monolayers (SAMs) formed electrode is very attractive subject in bioelectrochemistry. This paper reports a real-time visualization of cyt *c* adsorption processes on an 11-mercaptoundecanoic acid-modified Au electrode together with simultaneous electrochemical measurements. Adsorbing cyt *c* molecules were observed on a subsecond time resolution simultaneously with increasing redox currents from cyt *c* using EC-HS-AFM. The root mean square roughness (*R*_RMS_) from the AFM images and the number of the electrochemically active cyt *c* molecules adsorbed onto the electrode (*Γ*) simultaneously increased in positive cooperativity. Cyt *c* molecules were fully adsorbed on the electrode in the AFM images when the peak currents were steady. This use of electrochemical HS-AFM significantly facilitates understanding of dynamic behavior of biomolecules on the electrode interface and contributes to the further development of bioelectronics.

## Introduction

Understanding dynamic protein behavior on the electrode interface could enhance the efficiency of developing bioelectronics devices, such as biosensors and biofuel cells. To understand the correlation between molecular dynamic behavior and the electrochemical response of proteins adsorbed on the electrode, numerous studies have used spectro-electrochemical techniques that combine electrochemical measurements and various spectroscopy techniques, such as surface-enhanced resonance Raman (SERRS) [1–3] and surface-enhanced infrared absorption (SEIRA) [4–6]. Electrochemical quartz crystal microbalance (EQCM) and surface plasmon resonance (SPR) measurements also have been used to study dynamic molecular adsorption processes [7–9]. These techniques provide excellent opportunities to study redox protein mechanisms and provide overall information concerning the interface between proteins and an electrode; however, the local structure is not obtained, due to a lack of spatial resolution. Moreover, sometimes spectroscopic approaches suffer from the limitation where only protein chromophores are observed. Thus far, ex- or in-situ AFM imaging with electrochemical measurements have been employed to characterize protein adsorption properties on electrodes [10–12]. Furthermore, integrated AFM and scanning electrochemical microscopy (SECM) were developed to obtain in-situ information on electrochemical process at a liquid-solid interface [13, 14]. However, due to a limitation of temporal resolution of conventional AFM, it was difficult to directly monitor rapid protein adsorption processes and the relevant electrochemical processes. In contrast, high-speed atomic force microscopy (HS-AFM) was recently established, which allowed us to directly visualize several dynamic protein events, such as structural changes, molecular interactions, diffusion processes, and molecule adsorption onto a solid surface [15–20].

In this paper, we report the development of an electrochemical AFM based on HS-AFM, which was constructed in the laboratory and can analyze electrochemical measurements with the advantage of fast imaging. This approach could allow direct visualization of dynamic biological molecule behavior at the electrode with simultaneous electrochemical measurements. We applied electrochemical HS-AFM to observe the cytochrome *c* (cyt *c*) electrochemical reaction and adsorption processes on a self-assembled monolayers (SAMs)-modified Au electrode surface. Cyt *c* plays a crucial role as an electron transfer protein in eukaryotic and many prokaryotic respiratory chains. Cyt *c* is the most extensively studied protein in bioelectrochemistry [21] because cyt *c* is often regarded as a model metalloprotein for studying electron transfer within and between proteins. It is well known that cyt *c* electrostatically immobilizes on SAMs of carboxylic acid-terminated alkanethiols via lysine residues surrounding the cyt *c* heme crevice and exhibits a reversible voltammetric response [22–27]. Numerous studies on direct electrochemistry of cyt *c* adsorbed on a SAM-modified [25] electrode have employed various surface analysis techniques [5, 9, 11, 28–33]. Until now, however, cyt *c* molecules adsorbed on electrodes have not been directly observed through real-time imaging using microscopy techniques. This study demonstrates, to our knowledge, for the first time real-time visualization of cyt *c* adsorption processes on an 11-mercaptoundecanoic acid (MUA)-modified Au electrode with simultaneous electrochemical measurements.

## Materials and Methods

### Preparation of the SAM-modified gold electrode

A gold (111) substrate was used as the working electrode. The gold was deposited on a freshly cleaved mica substrate through vapor deposition and immersed in a 1 μM ethanol solution composed of MUA (Sigma) for 16 h at room temperature after flame annealing. The resulting gold (111)-mica substrates were thoroughly rinsed with ethanol and a 10 mM sodium phosphate buffer solution (pH 7.0) to remove the physically adsorbed thiol molecules. The SAM-modified gold (111)-mica substrate was affixed to the HS-AFM glass sample stage using an electricity-conducting adhesive.

### The electrochemical HS-AFM measurements

We used a laboratory-constructed high-speed AFM apparatus [15, 16]. The cantilevers (Olympus) used were 6–7 μm long, 2 μm wide, and 90 nm thick. The spring constant was 0.1–0.2 N/m, and the resonant frequency and quality factor in an aqueous solution were 0.7–1 MHz and ~2, respectively. For AFM imaging, the free oscillation amplitude was ~2 nm, and the set-point amplitude was approximately 90% of the free oscillation amplitude. An amorphous carbon tip was grown on the original tip through electron-beam deposition. For simultaneous electrochemical measurements, a Pt wire counter electrode and Ag wire reference electrode were incorporated into the HS-AFM cantilever holder (S1 Fig.). The electrochemical experiments were conducted using an ALS electrochemical analyzer (Model 1200b). The potentials cited in this paper refer to the Ag wire electrode with the potential −150 mV vs. Ag/AgCl (3 M NaCl) in 10 mM sodium phosphate buffer solution (pH 7.0). We performed the experiment to determine the potential of the Ag wire vs. Ag/AgCl by using 2,6-dichlorophenolindophenol. This experiment was conducted under equal conditions (10 mM sodium phosphate buffer solution at pH 7.0). The current densities were calculated with respect to the geometric electrode area 1.76 × 10^−6^ m^2^ (1.5 mm diameter gold (111)-mica substrates). The measurements were collected at room temperature. Before the measurements, oxygen was purged from the buffer solution through bubbling with highly purified nitrogen for 30 min. The electrochemical HS-AFM (EC-HS-AFM) measurements began when a 0.5 μL aliquot of 1 mM cyt *c* from horse heart (Sigma, USA) was added to a 10 mM sodium phosphate buffer solution (pH 7.0) at the total volume 70.5 μL. The final cyt *c* concentration was 7.1 μM.

## Results and Discussion

Cyclic voltammetric measurements using our newly constructed EC-HS-AFM were performed to confirm the redox behavior of the 0.1 mM 2,6-dichlorophenolindophenol (DCPIP) in 50 mM pH 7.0 HEPES buffer with a gold electrode. Because reversible redox peaks from DCPIP were observed in a diffusion system, EC-HS-AFM was established as the potential control for the sample stage.

**Figure 1:**
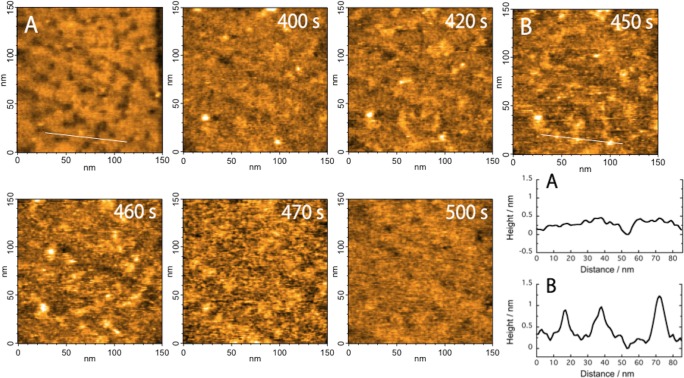
AFM images show (A) the MUA-modified gold surface and (B) cyt *c* adsorbed on the MUA SAM at 450 sec. Continuous AFM images of adsorbing cyt *c* molecules with real-time labels. Frame rate, 2 frames/s; image area, 150 × 150 nm^2^. The cross sections of the images in (A) and (B), along the short white line, are shown in the lower right.

Fig. 1A shows an AFM image of the MUA-modified gold electrode and a cross-section profile with an arbitrarily configured line in the image. The SAM-modified gold surface was flat with a well-packed monolayer and confirmed small pits. This image was consistent with typical AFM images of 11-mercaptoundecanol or MUA SAMs on a Au (111) substrate [34]. The EC-HS-AFM measurements were collected on the MUA-modified gold electrode as the sample stage after cyt *c* was injected into the analytical solution. Fig. 1 presents AFM images of cyt *c* molecules adsorbing on the MUA-modified gold surface collected at 2 frame s^−1^ (see S1 movie). In S1 movie, the cyt c molecules began to adsorb on the MUA-modified gold surface approximately 340 s after injection. The data generated from the time cyt *c* was added through the beginning of adsorption might be due to cyt *c* molecules approaching the nearby surface through diffusion. Randomly adsorbing cyt *c* molecules on the electrode were gradually observed from 340 s to 430 s in S1 movie. Cyt *c* molecules also frequently disappear immediately after adsorption during this period, which suggests that cyt *c* molecules rapidly move onto the surface when a small number of molecules adsorb. Next, rapid and remarkable cyt *c* molecule adsorption was observed from 430 s to 490 s followed by a full adsorption on the electrode surface after 500 s. After 500 s, no noticeable morphologically changes were observed in the AFM images likely because the electrode was full covered by molecules, and the cyt *c* molecules did not form multilayers.

**Figure 2:**
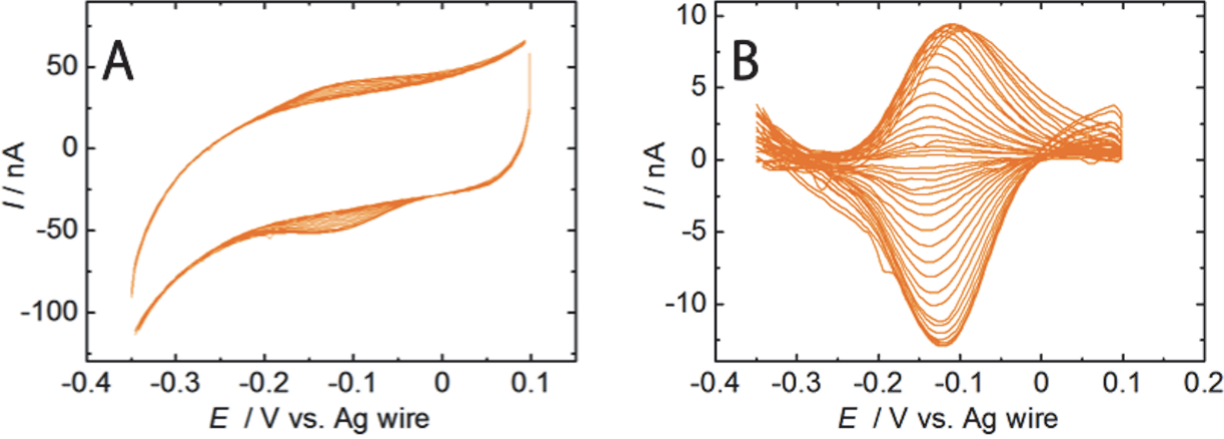
CVs of cyt *c* adsorbed on the electrode. (A) CVs of cyt *c* molecules adsorbed on the MUA electrode in a 10 mM phosphate buffer solution (pH 7.0) from 333 to 522 s (from −0.35 V to 0.1 V, each segment is 4.5 s). (B) Background-subtracted CVs from the voltammogram from 313 to 324 s and 333 to 522 s (from inside to outside, each segment is 4.5 s). The voltammograms were collected at a scan rate of 100 mVs^−1^. Ag wire was used as a reference electrode.

Cyclic voltammograms (CVs) were sequentially collected while cyt *c* adsorption was monitored using EC-HS-AFM and are shown from 337.5 s to 517.5 s in Fig. 2. CVs for saturated cyt *c* adsorption on the gold electrode with an MUA SAM showed the cathodic and anodic peak potentials −122 and −102 mV (vs. Ag wire), respectively, at 522 s. The cyt *c* midpoint potential was approximately −112 mV (vs. Ag wire). As depicted in Fig. 2, current redox peaks for cyt *c* were also observed and increased from 340 s to 480 s simultaneously with the cyt *c* adsorption processes in the AFM images. The peak currents were steady after 500 s. An EC-HS-AFM experiment of the SAM unmodified gold electrode (bare gold surface) was conducted for comparisons of the MUA-modified gold electrode (see S2 movie and S2 Fig.).

**Figure 3:**
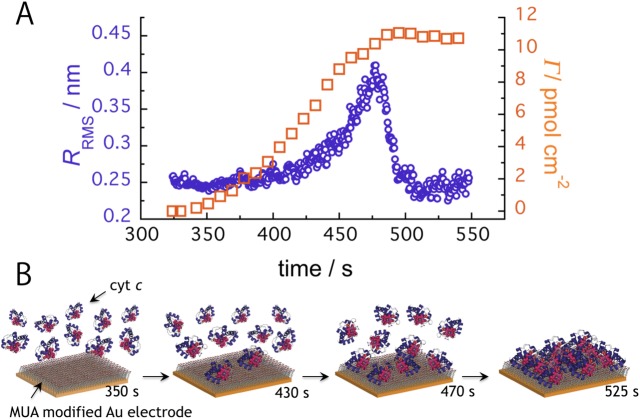
Time-course analysis of *R*_RMS_ and *Γ* values. (A) Time evolution of *R*_RMS_ values from the HS-AFM images (open circle) and the level of electrochemically active cyt *c* (*Γ*) from the cyclic voltammograms (open square). (B) Schematic of the adsorbing cyt *c* molecules on the MUA-modified electrode at each time point.

The root mean square (RMS) roughness *R*_RMS_, which is given by the standard deviation of height, for the commonly used AFM images were used as roughness profile ordinates and plotted against time in Fig. 3A. Fig. 3B shows a schematic explanation of the cyt *c* adsorption processes in Fig. 3A and S1 movie. The *R*_RMS_ variations indicate that cyt *c* adsorbed on the electrode. The *R*_RMS_ values exponentially increased beginning at approximately 430 s and peaked at 470 s. At approximately the *R*_RMS_ peak time, cyt *c* adsorption was not saturated, but cyt *c* covered almost half of the sorbable sites on the electrode surface. After 470 s, the *R*_RMS_ values immediately decreased because more cyt *c* molecules adsorbed more on the electrode surface, which likely filled a gap between the immobilized cyt *c* molecules. We simulated the evolution time for the *R*_RMS_ values from random adsorption on a 50 × 50 site based on a change in the number of adsorbing molecules in proportion to the number of the active adsorption sites. The simulation model results show that *R*_RMS_ values increase logarithmically with increased surface coverage and reach a maximum at almost half of the coverage (see S3 movie). In Fig. 3A, it is noteworthy that the *R*_RMS_ values an exponentially-increased with time, which indicates that the cyt *c* adsorption process has a cooperative effect. This result suggests that the cooperative effect includes potential interactions, such as electrostatic or hydrophobic interactions between the immobilized cyt *c* molecules. Reports show that protein adsorption is a cooperative process [35, 36]. The slight change in *R*_RMS_ values from approximately 500 s to 550 s indicates saturated cyt *c* adsorption. We conclude that this result supports protein monolayer formation on the MUA-modified gold electrode as suggested in the AFM S1 movie. The number of the electrochemically active cyt *c* molecules adsorbed onto the electrode (*Γ*) was estimated by integrating the observed CV curve reduction peak using the following equation: *Γ = Q/nFA. Q* is the charge involved in the reaction, *n* is the number of electrons involved in the redox process (for cyt *c*-Fe(II) oxidation, *n* = 1), *F* is the Faraday constant, and *A* is the electrode surface area. As depicted in Fig. 3A, the time dependence of the *Γ* values was also plotted using a sigmoid and the *R*_RMS_ values; these results suggest cooperative cyt *c* adsorption. The value *Γ* at the steady state after 500 s was calculated as 11 pmol cm^−2^ (the largest value in several times), which is consistent with the experimental and theoretical values for full monolayer coverage at 13 pmol cm^−2^ [24, 29]. This value also agrees with that of the integrated the peak currents from CV, 9.1 pmol cm^−2^ [27]. In addition, Nakano et al. showed that the QCM analysis and AFM imaging supported the cyt *c* monolayer formation on the MUA surface [11]. The finding of the cyt *c* monolayer formation agrees with our results of CV and HS-AFM experiments. The difference between our results and their report on the value of electroactive surface coverage could be explained as the way of cyt *c* immobilization. The cyt *c* was covalently immobilized on MUA surface in their report, while it was immobilized by the electrostatic interaction in our case. The QCM analysis in their report showed that the value of apparent cyt *c* surface concentration was 28 ± 12 pmol cm^−2^, which was larger than those calculated from CVs (7.2 ± 4.8 pmol cm^−2^) in their report and our data (11 pmol cm^−2^). In the case of the QCM analysis, Furusawa et al. demonstrated that apparent larger frequency changes by the protein immobilization onto the gold surface in the aqueous solutions (Δ*F*_water_) were observed compared with those in the dry air phases (Δ*F*_air_) owing to the interaction with the surrounding water molecules [37]. Myoglobin (Mw: 16.9 kDa), for example, exhibited apparent mass ratio 2.1 [(−Δ*F*_water_)/(−Δ*F*_air_)]. It could happen in the case of cyt *c*.

The *Γ* values compared with the *R*_RMS_ in Fig. 3A show that both parameters simultaneously increased until 470 s. At approximately 500 s, the *Γ* values reached the maximum and the *R*_RMS_ was returned to the initial value, respectively. On the other hand, although the *Γ* values began to increase at approximately ~400 s, the *R*_RMS_ increased after ~400 s. This observation is presumably due to unimmobilized cyt *c* molecules on the electrode that were not imaged using HS-AFM because the movement was too rapid, but these molecules which are located adjacent the electrode could exhibit the electrochemical response.

In addition, the peak potential positively shifted with the increasing *Γ* values (S3 Fig.). A change in cyt *c* redox potential is induced by several factors, the heme axial ligands [38], the heme environment polarity [39], and the heme solvent accessibility [40] as well as due to the electrostatic interactions between heme propionates and amino acid residues [41, 42]. Recent research has shown that heme propionate hydration shifts the reduction potential of approximately 50 mV toward more positive values [42]. When there is more hydrophobicity and a localized structural change due to well-packed cyt *c* adsorption on the electrode with MUA SAM, a positive cyt *c* redox potential shift should be observed that originates with the environment surrounding the heme. The localized structural change of the environment surrounding the heme could modulate several factors that affect redox potential. Further studies are necessary to discern the heme conditions using spectroscopic techniques.

**Figure 4:**
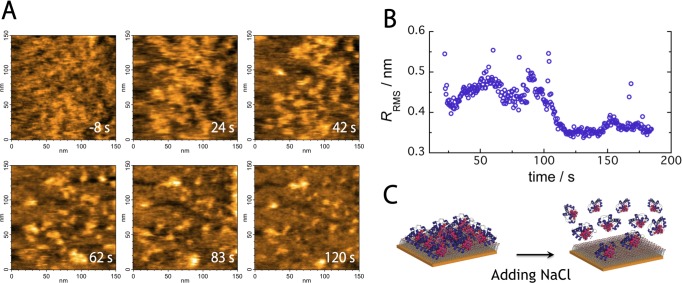
Real-time cyt *c* desorption processes from the MUA-modified gold electrode. (A) Continuous AFM images of desorbing cyt *c* molecules. Frame rate, 2 frames/s; image area, 150 × 150 nm^2^. (B) Time evolution of *R*_RMS_ values at the higher ionic strengths. (C) Schematic of desorbing cyt *c* molecules from the MUA-modified electrode.

For cyt *c* adsorption on the saturated electrode, 20 μL of the electrolyte solution of EC-HS-AFM was extracted, and 20 μL of a 4 M NaCl solution was injected into the analytical solution (NaCl at the final concentration 1.13 M) to generate an analytical solution with a high salt concentration. Cyt *c* molecules desorbed from the MUA-modified gold electrode surface immediately after adding the NaCl solution as shown Fig. 4A and S4 movie. Most of the cyt *c* molecules desorbed, and the surface with exposed MUA SAMs was observed after 120 s. Similarly, the *R*_RMS_ decreased at approximately 120 s but was steady after 120 s as shown Fig. 4B. Certain ordinates of the points were unusual as shown in Fig. 4B; they are due to spike-like noise in AFM images with an abrupt increase in ionic strength. Fig. 4C shows a schematic representation of the desorbing cyt *c* molecules on the MUA SAM-modified electrode. The results indicate that cyt *c* molecules desorb due to weaker electrostatic adsorption on electrode-coated MUA SAMs at higher ionic strengths.

## Conclusions

In summary, we altered the HS-AFM sample stage to control the electrical potentials and constructed EC-HS-AFM equipment that simultaneously allows direct visualization of dynamic behavior with nanoscale resolution and electrochemical measurements. Using this EC-HS-AFM approach, this study demonstrates, to our knowledge, for the first time direct observation of adsorption processes and the cyt *c* electron transfer reaction on a SAM-modified gold electrode in real-time. Cyt *c* molecules were fully adsorbed at approximately 160 s with increasing redox currents from cyt *c*. The details of cooperative adsorption have not been clarified; however, the findings herein could lead to new, in-depth discoveries on the cyt *c* adsorption processes. EC-HS-AFM also enables us to directly visualize the structure and dynamics of molecules in response to an applied potential. This use of EC-HS-AFM significantly facilitates studies aimed at understanding the dynamic behavior of biomolecules on an electrode interface and contributes to the further development of bioelectronics.

## Supporting Information

S1 Fig Schematic of the EC-HS-AFM cantilever holder and electrodes. (TIF)

S2 Fig CVs of the bare gold electrode in a 10 mM phosphate buffer solution (pH 7.0) containing cyt c (final concentration of 41 μM). CVs were synchronized with S2 movie. The voltammograms were collected from 0 to 600 s (from −0.4 V to 0.2 V, each segment is 6 s) at a scan rate of 100 mVs^−1^.
(TIF)

S3 Fig Time evolution of the cyt *c* (closed circle) cathodic peak potential and surface coverage of electrochemically active cyt *c* (*Γ*) (closed square). (TIF)

S1 Movie HS-AFM images of cyt *c* adsorbing on the MUA-modified electrode (left). x/y = 150/150 nm, 10-fold speed. Cyt *c* was added (0 s) at a final concentration of 7.1 μM. A synchronized CV with the HS-AFM images of cyt *c* adsorbing on the MUA-modified electrode in a 10 mM phosphate buffer solution (pH 7.0) from 315 to 544 s (right). At a scan rate of 100 mVs^−1^.
(AVI)

S2 Movie HS-AFM images of the bare gold electrode. x/y = 150/150 nm, 10-fold speed. Initially, 1 μL of cyt *c* (1 mM) was injected at 0 s and then 2 μL of cyt *c* (1 mM) was injected at 126 s (final concentration of 41 μM).
(AVI)

S3 Movie The simulated movie is a time evolution of *R*_RMS_ values. The inset movie comprises images of random adsorption on a 50 × 50 site.
(AVI)

S4 Movie HS-AFM images of cyt *c* desorbing from the MUA-modified electrode. x/y = 150/150 nm, 10-fold speed, total time 184.5 s. NaCl was added (0 s) at a final concentration of 1.13 M.
(AVI)
