# Supplementary figures and images for: Real-Time Dynamic Adsorption Processes of Cytochrome c on an Electrode Observed through Electrochemical High-Speed Atomic Force Microscopy
Source: PLoS One. 2015 Feb 11;10(2):e0116685. doi: 10.1371/journal.pone.0116685 (PMC4324961; doi:10.1371/journal.pone.0116685)

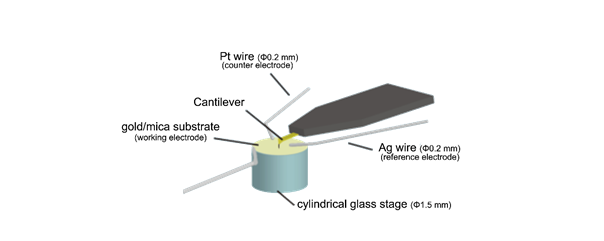

Supplement: S1 Fig — (TIF) [file pone.0116685.s001.tif]

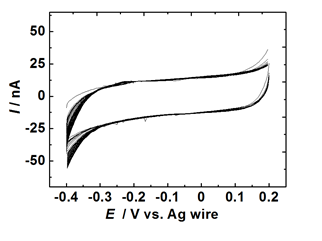

Supplement: S2 Fig — CVs were synchronized with S2 movie. The voltammograms were collected from 0 to 600 s (from −0.4 V to 0.2 V, each segment is 6 s) at a scan rate of 100 mVs−1. (TIF) [file pone.0116685.s002.tif]

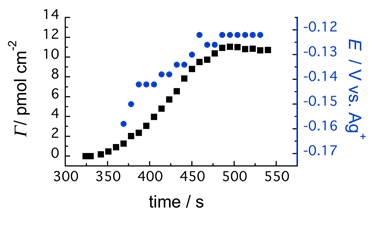

Supplement: S3 Fig — (TIF) [file pone.0116685.s003.tif]
